# Use of Extrinsic Motivators to Improve the BMI of Obese or Overweight Adolescents: Systematic Review

**DOI:** 10.2196/57458

**Published:** 2024-12-30

**Authors:** Ana Gonçalves, Pedro Augusto Simões, Bernardo Sousa-Pinto, Tiago Taveira-Gomes

**Affiliations:** 1 Faculty of Medicine Universidade do Porto Porto Portugal; 2 Faculty of Health Sciences University of Beira Interior Covilha Portugal; 3 Health Sciences Research Centre - University of Beira Interior University of Beira Interior Covilha Portugal; 4 Department of Community Medicine Information and Health Decision Sciences Faculty of Medicine of the University of Porto Porto Portugal; 5 Centre for Health Technology and Services Research Health Research Network Faculty of Medicine of the University of Porto Porto Portugal

**Keywords:** adolescents, obesity, overweight, extrinsic motivators, body mass index

## Abstract

**Background:**

The prevalence of overweight and obesity is increasing at an alarming rate in children and adolescents worldwide. Given the dimensions of the problem, the treatment of childhood obesity is considered extremely important. Current evidence indicates that behavioral and cognitive behavioral strategies combined with diet and physical activity approaches may assist in reducing adolescent obesity.

**Objective:**

The purpose of this systematic review was to evaluate the use of extrinsic motivators for improving the BMI of obese or overweight adolescents.

**Methods:**

The inclusion criteria were as follows: (1) overweight or obese adolescents, (2) intervention using extrinsic motivators, and (3) outcome variables related to weight status. The exclusion criterion was the presence of an associated chronic disease. The search process was conducted in PubMed and Web of Science (last searched on April 23, 2023). The risk of bias was evaluated independently by 2 authors using Cochrane tools (RoB2 [randomized controlled trials], ROBINS-I, and ROBINS-E).

**Results:**

From 3163 studies identified, 20 articles (corresponding to 18 studies) were included in the analysis. The studies differed in terms of study design, sample size, follow-up duration, outcomes reported, and extrinsic motivators used. Most of the studies had videogames or apps as interventions. Of the 18 studies, 9 (50%) reported a statistically significant decrease in BMI. The most used extrinsic motivators were “motivation” (n=13), “feedback” (n=10), and “rewards” (n=9). Among the motivators, “reminders” (100%) and “peer-support” (80%) appeared to have high impacts on BMI reduction.

**Conclusions:**

The heterogeneity of the included studies made analysis difficult. No study evaluated extrinsic motivators in isolation. Most of the studies had a moderate or high risk of bias. The extrinsic motivators “reminders” and “peer-support” appeared to be useful. Further studies are needed, and these should include well-designed randomized controlled trials and studies involving homogeneity in BMI measures, consistent extrinsic motivator definitions, and longer durations to better understand the long-term impacts of extrinsic motivators on weight management success.

## Introduction

The prevalence of overweight and obesity is increasing at an alarming rate in children and adolescents worldwide, and the rise has occurred similarly among both boys and girls [1,2]. Over 340 million children and adolescents aged 5-19 years were overweight or obese in 2016 [2]. In the United States, in 2020-2021, 17% of children and adolescents aged 10-17 years had obesity, with rates significantly higher for non-Hispanic Black (22.9%), Hispanic (22.4%), and non-Hispanic American Indian/Alaska Native (20.5%) individuals [3]. Similarly, in China, in 2020, the prevalence of overweight (including obesity) was 19% for children aged 6-17 years [4], and in Europe, in 2018, 19% of children aged 15 years were either overweight or obese [5]. The data available in Portugal are different, with a prevalence of overweight (including obesity) between 20% and 40% [6].

The widespread obesity among young people is generating significant social concern for public health, including not only the number of children affected but also the consequences [7]. A raised BMI is a major risk factor for cardiovascular diseases, diabetes, musculoskeletal disorders, and some cancers, and the risk for these diseases increases with an increase in BMI [2]. Obesity also has psychological consequences, such as low self-esteem, sadness, nervousness, and a negative self-image [7]. Overweight and obesity, as well as their related diseases, are largely preventable [2]. The global prevalence of obesity in children and adolescents almost doubled between 1980 and 2015 [8]. In view of the high and increasing prevalence, an early and effective intervention is urgently required [9].

Adolescence is a vulnerable period for the development of obesity, and adolescent weight tracks strongly into adulthood. Adolescence is also a unique period of development, with puberty resulting in considerable physical, hormonal, and psychosocial changes, which need to be considered when evaluating the efficacy of treatment programs [10]. 

Given the dimensions of the problem, the treatment of childhood obesity is considered extremely important. Fundamental approaches involve changing food intake and quality, increasing effort in physical activity, and reducing sedentary habits [7]. Current evidence indicates that behavioral and cognitive behavioral strategies combined with diet and physical activity approaches may assist in reducing adolescent obesity [10].

According to the self-determination theory, extrinsic motivation is a type of motivation in which the behavior is a means of achieving external outcomes. There are 4 different types of extrinsic motivations, which vary on a spectrum according to the degree of internalization and autonomy: external regulation (motivated by external controls prescribed by others, like gaining rewards or avoiding punishment); introjected regulation (motivated by internal pressure from internalized constructs of external controls, such as feeling self-approval or avoiding feeling guilty); identified regulation (motivated because behavior is perceived as important and useful, like exercise to get healthy or lose weight); and integrated regulation (motivated because the behavior is in concordance with one’s values and sense of self, such as feeling of identification) [11]. Some examples of extrinsic motivators are rewards/points, avatars, challenges, and feedback [11-13].

To our knowledge and research, no review has studied extrinsic motivators as a whole in weight loss among obese adolescents. Most studies have analyzed the impact of specific interventions (eg, active video gaming) on weight or BMI. Byrne et al [14] found that motivation was also affected by feedback from a mobile app that involved caring for a virtual pet. Andrade et al [15] found that “owing to the fun, pleasure, joy, and innovative technology provided by exergames, they have the potential to attract children and adolescents who are overweight or obese.”

The purpose of this systematic review was to evaluate the use of extrinsic motivators for improving the BMI of obese or overweight adolescents.

## Methods

### Reporting Guidelines

This review has been reported according to the recommendations of the PRISMA (Preferred Reporting Items for Systematic Reviews and Meta-Analyses) guidelines.

### Eligibility Criteria

The review question was framed using the PICO framework, and the following eligibility criteria were used: (1) Population: overweight (P85-95 for age and gender) or obese (≥P95 for age and gender) adolescents (age range 10-19 years; according to the World Health Organization [WHO]); (2) Intervention: extrinsic motivators; (3) Comparison: usual care or other interventions; and (4) Outcome: variables related to weight status (weight or BMI z-scores).

We only included published primary research studies in Portuguese, Spanish, English, French, and Italian. The exclusion criterion was presence of associated chronic disease. 

### Information Sources

The search process was conducted in PubMed and Web of Science (last searched on April 23, 2023).

### Search Strategy

The databases PubMed and Web of Science were searched using the following terms: adolescent (a validated search filter) [16], obesity, overweight, pediatric obesity, weight loss, gamification, and telemedicine.

The search on PubMed was carried out for the title, abstract, and MeSH terms, and the search on Web of Science was carried out for the title, abstract, and keywords. The full strategy used is presented in Multimedia Appendix 1.

The references of the included studies and other reviews were analyzed for eligibility.

### Selection Process

Titles and abstracts identified from the search were transferred to the site “Rayyan” for the relevance assessment process and deduplication. Potentially eligible studies were first screened independently by 2 authors (AG and PAS) according to the title and abstract to evaluate if they met the inclusion criteria. Then, the full texts were read independently by the same 2 authors, and a decision was made about inclusion in the review. Disagreements were resolved by consensus among the authors.

### Data Collection Process

The data were collected from each report independently by 2 authors (AG and PAS) with Excel (Microsoft Corp). Disagreements were resolved by consensus among the authors. A set of variables was chosen from consensus among all authors, and the following data were systematically extracted for each eligible article: bibliographic information, study design, target population, inclusion criteria, exclusion criteria, sample size, demographic characteristics, duration of the study/follow-up/time of data collection, purpose, intervention, extrinsic motivator, and outcome BMI.

### Study Risk of Bias Assessment

The risk of bias was evaluated with Cochrane tools: RoB2 for randomized controlled trials (RCTs), ROBINS-I for interventional studies, and ROBINS-E for exposure studies. Two authors (AG and PAS) independently assigned the risk of bias to each study, and disagreements were resolved by consensus among the authors. 

## Results

### Included Studies

In total, 3163 studies were identified from the searched databases, and after removing duplicates, 2653 articles were left for the following steps. Moreover, 2558 articles were deleted after analyzing the title and abstract. Among the remaining articles, 92 were retrieved for more detailed assessment (3 articles could not be retrieved after contacting the authors), and they were evaluated considering the inclusion and exclusion criteria. After the evaluation of the full text, 72 articles were excluded for not meeting the inclusion criteria (population, intervention, outcome, and language). Finally, 20 articles (corresponding to 18 studies) met the inclusion criteria and were included in the analysis [17-36].

**Figure 1:**
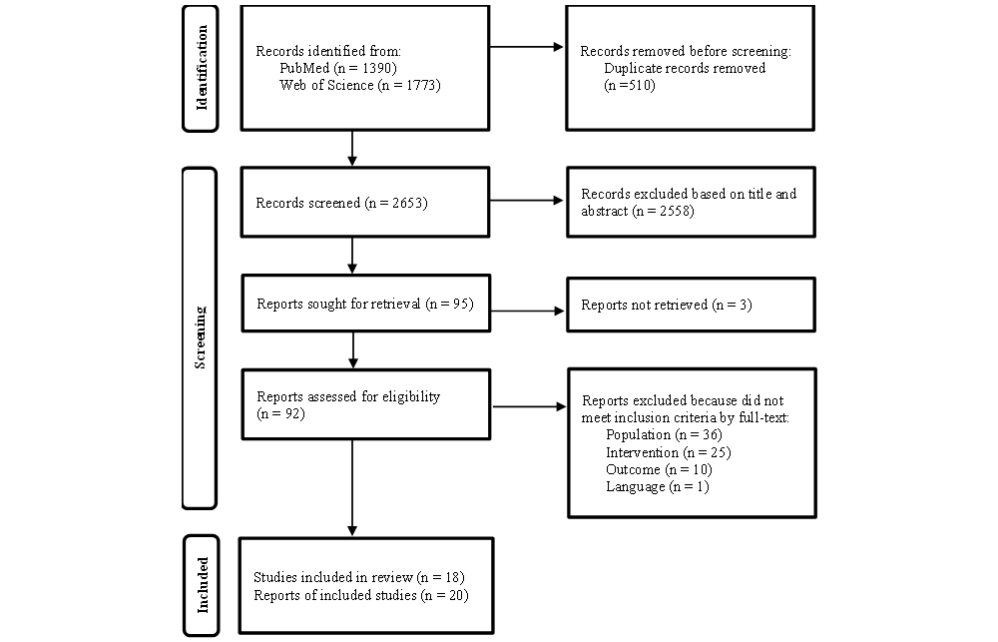
Flow diagram showing the article selection process.

Figure 1 shows the steps of the study selection process following the PRISMA statement. The PRISMA checklist is presented in Multimedia Appendix 2.

### Study Characteristics

The studies included were published between 2006 and 2022 (12 in the last 10 years) and were performed in 8 different countries: 11 in the United States, 1 in China, 1 in Singapore, 1 in Korea, 1 in Canada, 1 in Switzerland, 1 in Italy, and 1 in New Zealand.

### Risk of Bias in the Studies

**Figure 2:**
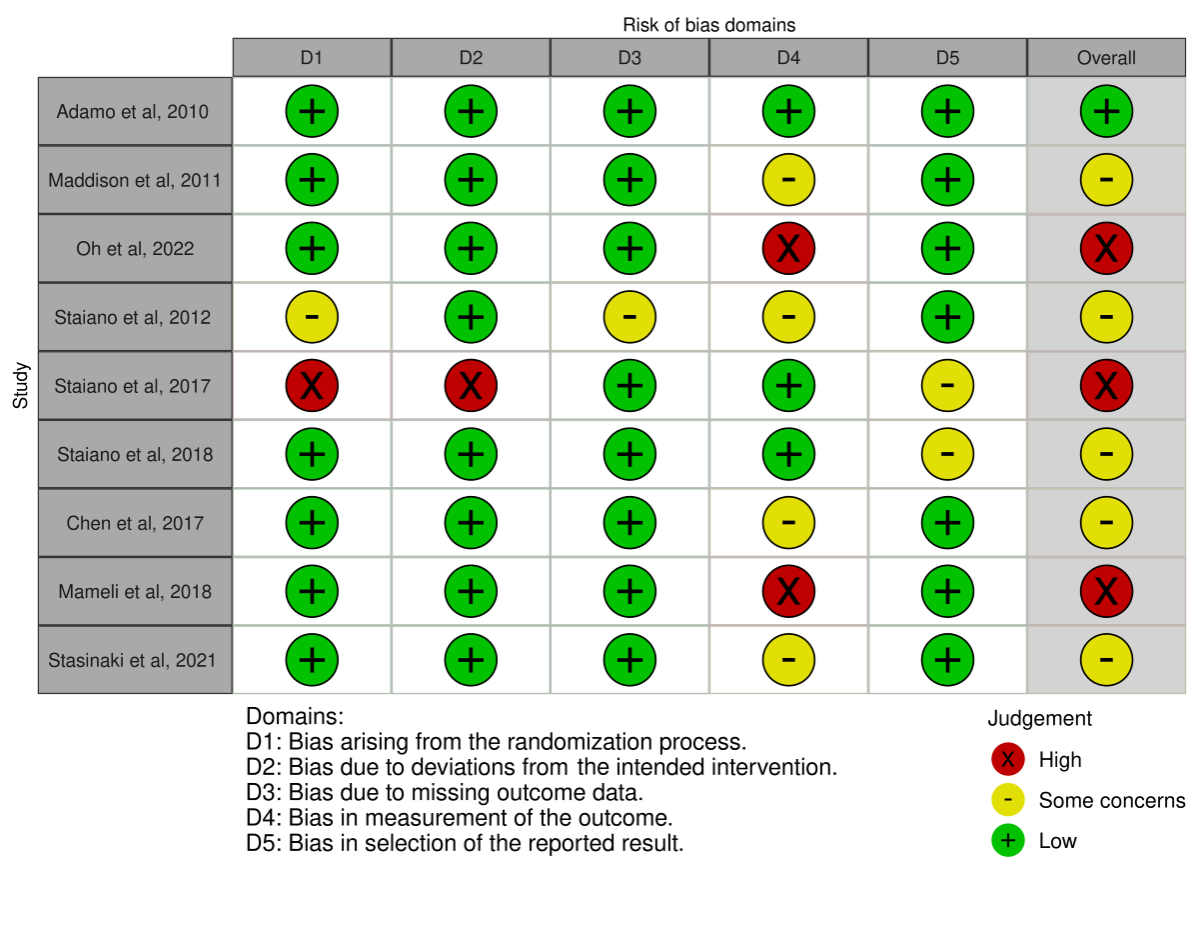
Risk of bias according to the ROB-2 tool.

**Figure 3:**
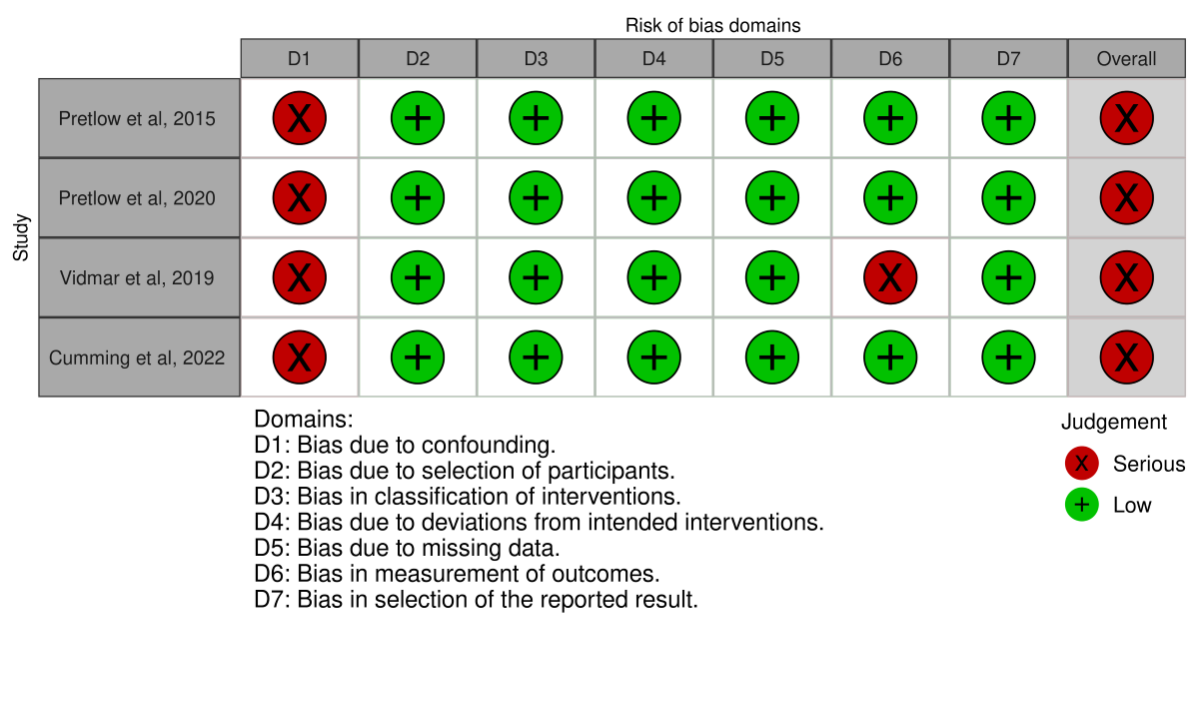
Risk of bias according to the ROBINS-I tool.

**Figure 4:**
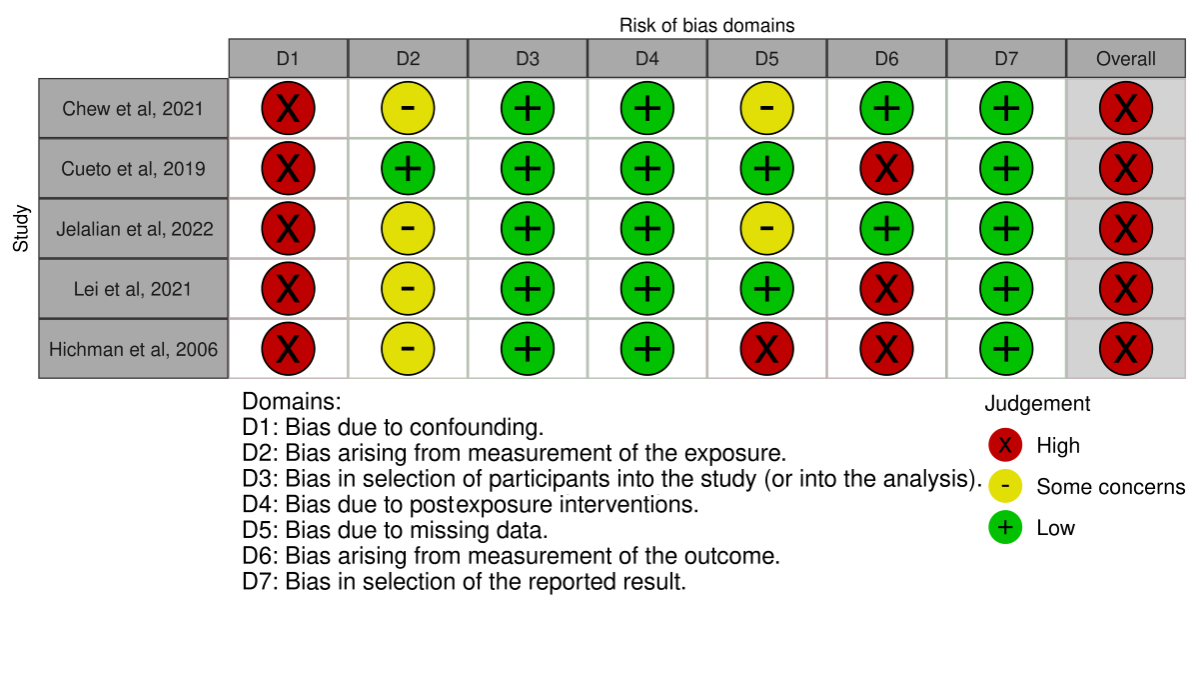
Risk of bias according to the ROBINS-E tool.

There was heterogeneity in the design of the studies: 13 studies were clinical trials (9 RCTs), 4 were cohort studies, and 1 was a retrospective observational study. Detailed findings concerning the risk of bias according to the ROB-2, ROBINS-I, and ROBINS-E tools are presented in Figure 2 [17-23,28,31], Figure 3 [29,30,32,33], and Figure 4 [24-27,34], respectively. Most of the RCTs presented a moderate risk of bias, and all other study designs had a high or serious risk.

### Results of Individual Studies

The characteristics of the included studies are summarized in Table 1.

Regarding the sample size, we verified that 14 studies had less than 100 participants (minimum 24 and maximum 85), 1 had 322 participants, and 3 had more than 1000 participants (minimum 1120 and maximum 3500). The follow-up of the studies varied from 3 weeks to more than 18 months. However, most of the studies had a follow-up of 3 to 6 months.

**Table 1:** Main characteristics of the selected studies.

Author, year, and country	Study design	Target population	Subjects	Follow-up	Intervention	Control	Extrinsic motivator
Adamo et al [17], 2010; Canada	RCT^a^	IC^b^: 12-17 years and pBMI^c^ >95 or pBMI >85 with high risk of type 2 diabetes or cardiovascular disease EC^d^: Medication or supplement that affects body composition, regular physical activity, psychiatric illness, and contraindication to physical activity	Sample: 26 (13 I^e^, 13 C^f^) Gender: 53.8% (I, C) female Mean age: 13.9 (SD 1.4) (I), 15.1 (SD 1.8) (C) years pBMI: 97.9 (SD 1.5) (I), 97.8 (SD 2.7) (C) Ethnicity: Not mentioned	2.5 months	Active videogame cycling	Stationary cycling to music	Feedback Select games
Maddison et al [18], 2011; New Zealand	RCT	IC: 10-14 years and overweight/obese EC: Own active videogames or contraindication for physical activity	Sample: 322 (160 I, 162 C) Gender: 72.5% (I), 73.5% (C) male Mean age: 11.6 (SD 1.1) years (I, C) zBMI^g^: 1.3 (SD 1.1) (I, C) Ethnicity: 57.5% (I), 56.2% (C) NZ^h^ European	6 months	Active videogame	Nonactive videogames	Avatar Select games
Oh et al [19], 2022; Korea	RCT	IC: 10-17 years and pBMI >85 EC: Pharmacologic treatment, sign of hormonal/orthopedic/metabolic/cardiovascular illness, and physical activity outside school	Sample: 24 (12 I, 12 C) Gender: 83.33% (I, C) male Mean age: 13.58 (SD 1.44) (I), 13.08 (SD 2.35) (C) years BMI: 24.99 (SD 1.35) (I), 25.03 (SD 1.92) (C) Ethnicity: Not mentioned	3 weeks	Active videogame SUIKA (Super Kids Adventure)	Active videogame NINS (Nintendo Switch “Ring Fit Adventure”)	Avatar Feedback Reminder Rewards
Staiano et al [20], 2012; United States	RCT	IC: African American, 15-19 years, and pBMI ≥75 EC: Not mentioned	Sample: 54 (19 CP^i^, 19 CO^j^, 16 C) Gender: 55.6% female Mean age: Not mentioned pBMI: 94.7 (SD 6.0) Ethnicity: African American	5 months	Active videogame (Nintendo Wii Active): cooperative or competitive	Usual daily activities	Motivation Peer-support Rewards
Staiano et al [21], 2017; United States	RCT	IC: 14-18 years, pBMI ≥85, female, and postmenarcheal period EC: Pregnancy, contraindication for physical activity, recent hospitalization for mental illness, and inability to comply with study procedures	Sample: 41 (22 I, 19 C) Gender: 100% female Mean age: 15.3 (SD 1.2) (I), 16.1 (SD 1.4) (C) years pBMI: 97.4 (SD 2.9) (I), 97.1 (SD 3.3) (C) Ethnicity: 64% (I), 61% (C) African American	3.5 months	Active videogame	Self-directed care control	Motivation Rewards Select games
Staiano et al [22], 2018; United States	RCT	IC: 10-12 years and pBMI ≥85 EC: Pregnancy and contraindication for physical activity	Sample: 46 (23 I, 23 C) Gender: 54% male Mean age: 11.2 (SD 0.8) years zBMI: 2.06 (SD 0.46) (I), 2.10 (SD 0.42) (C) Ethnicity: 57% African American, 41% White	6 months	Active videogame	Usual level of physical activity	Challenges Feedback Motivation Select games
Chen et al [23], 2017; United States	RCT	IC: 13-18 years and BMI ≥P85 EC: Not mentioned	Sample: 40 (23 I, 17 C) Gender: 58% (I), 53% (C) male Mean age: 15 (SD 1.69) (I), 14.77 (SD 1.60) (C) years zBMI: 1.6 (SD 0.50) (I), 1.54 (SD 0.42) (C) Ethnicity: 90% Chinese American	6 months	App (iStart Smart for Teens online program)	Online program’s content with information from an app	Motivation
Chew et al [24], 2021; Singapore	Cohort	IC: 10-17 years and pBMI >85 EC: Secondary causes of obesity, parents are non-English speakers, and logistic problems	Sample: 40 Gender: 58% male Mean age: 13.8 (SD 1.7) years BMI: 80% obesity Ethnicity: 45% Chinese, 33% Malay	6 months	App (Kurbo)	No	Feedback Motivation
Cueto et al [25], 2019; United States	Cohort	IC: 5-19 years EC: pBMI <85 at baseline and data measurement errors	Sample: 1120 Gender: 68.04% female Mean age: 12.0 (SD 2.5) years pBMI: 96.6 (SD 3.1) Ethnicity: Not mentioned	4 weeks, 3-4 months, and 6 months	App (Kurbo)	No	Feedback Motivation
Jelalian et al [26], 2022; United States	Cohort	IC: 5-17 years and pBMI >85 EC: Missing data, follow-up age >240.5 months, and measurement errors	Sample: 3500 Gender: 71.3% female Mean age: 12.79 (SD 2.41) years BMI: 29.65 (SD 5.56); %BMIp95: 116.31 (SD 19.57) Ethnicity: Not mentioned	0-3 months, 3-6 months, 6-12 months, 12-18 months, and >18 months	App (Kurbo)	No	Feedback Motivation
Lei et al [27], 2021; China	Observational	IC: 10-17 years and pBMI ≥85 EC: Secondary causes of obesity, pharmacologic/cirurgic intervention for weight reduction, logistic problems, and outliers	Sample: 2825 Gender: 54.8% female Mean age: 14.4 (SD 2.2) years pBMI: 48% ≥97 Ethnicity: Not mentioned	4 months	App (MetaWell)	No	Challenges Feedback
Mameli et al [28], 2018; Italy	RCT	IC: 10-17 years, pBMI ≥95, and Caucasian ethnic group EC: Genetic/syndromic obesity, psychiatric disease, and any condition compromising the ability to walk	Sample: 30 (16 I, 14 C) Gender: 68.8% (I), 57.1% (C) male Mean age: 12.6 (SD 1.7) (I), 12.4 (SD 2.2) (C) years BMI-SDS^k^: 2.20 (SD 0.47) (I), 2.09 (SD 0.34) (C) Ethnicity: Caucasian	3 months	App (MeTeDa)	Mediterranean diet plus physical activity	Challenges Feedback Motivation
Pretlow et al [29], 2015; United States	Pilot clinical trial without control	IC: Child/adolescent with overweight/obesity EC: Motivation scores <50/100 and logistic problems	Sample: 43 Gender: 65.1% female Mean age: 16 (SD 0.43) years pBMI: 37.2% >98 Ethnicity: 83.7% White	5 months	App	No	Motivation Peer-support Rewards
Pretlow et al [30], 2020; United States	Clinical trial without control	IC: 10-20 years, pBMI ≥95, and motivation score >50/100 EC: Not mentioned	Sample: 35 Gender: 51.4% female Mean age: 13.8 (SD 0.52) years pBMI: 34.3% ≥99 Ethnicity: 65.7% Caucasian	4 months	App	No	Motivation Peer-support Reminder Rewards
Stasinaki et al [31], 2021; Switzerland	RCT	IC: 10-18 years, pBMI >97 or >90 with ≥1 risk factor or co-morbidity EC: Not controlled psychiatric disease, medication that affects weight, and participation in another obesity treatment program in the past year	Sample: 31 (18 I, 13 C) Gender: 61.1% (I), 53.8% (C) male Mean age: 12.6 (I), 13.7 (C) years BMI-SDS: 2.5 (I, C) Ethnicity: Not mentioned	12 months	App (PathMate2) + multicomponent behavior change intervention	Multicomponent behavior change intervention	Challenges Feedback Motivation Rewards
Vidmar et al [32], 2019; United States	Pilot clinical trial	IC: 12-18 years and YFAS-c^l^ + EC: Obesity comorbidities, psychiatric disease or developmental delay, and inability to read English	Sample: 35 (18 I, 17 C) Gender: 72.22% (I), 47.06% (C) female Mean age: 14.44 (SD 1.65) (I), 14.35 (SD 1.77) (C) years BMI: Not mentioned Ethnicity: 61.11% (I) vs 64.71% (C) Hispanic	6 months	App (W8Loss2Go)	EMPOWER program	Motivation Peer-support Rewards
Cummings et al [33], 2022; United States	Clinical trial without control	IC: 13-18 years and pBMI >90 EC: Contraindication for use scanner, psychiatric medication, pregnancy, comorbidities, and visual acuity that could not be corrected	Sample: 26 Gender: 54% male Mean age: 14.81 (SD 1.59) years pBMI: 97.07 (SD 1.85) Ethnicity: 93% White	3 months	Digital health program	No	Challenges Feedback Motivation Rewards
Hinchman et al [34], 2006; United States	Cohort	IC: 11-17 years, completion of Operation Zero in 2001-2002, at risk of overweight, and overweight EC: Not mentioned	Sample: 85 (43 I, 42 C) Gender: 56% (I), 45% (C) female Age: 56% (I), 57% (C) ≥13 years pBMI: 98% (I), 85% (C) >95 Ethnicity: Not mentioned	12 months	Operation Zero	Members who never attended Operation Zero	Challenges Peer-support Rewards

^a^RCT: randomized controlled trial.

^b^IC: inclusion criteria.

^c^pBMI: BMI percentile.

^d^EC: exclusion criteria.

^e^I: intervention.

^f^C: control.

^g^zBMI: BMI z-score.

^h^NZ: New Zealand.

^i^CP: competitive arm.

^j^CO: cooperative arm.

^k^BMI-SD: BMI standard deviation.

^l^YFAS-c: Yale Food Addiction Scale for Children.

Regarding the demographic characteristics of the participants, 10 studies had a sample composed mainly of female adolescents (1 of them had only female participants). The mean age reported by the studies varied from 11.2 to 16 years. Moreover, 11 studies reported information about ethnicity (8 in the United States, 1 in Italy, 1 in Singapore, and 1 in New Zealand), and the majority of participants were Caucasian and African American.

### Interventions of the Studies

Adamo et al [17] assessed an intervention that involved 60-minute sessions 2 times per week with a GameBike interactive video gaming system (with a handlebar-mounted game controller) interfaced with a Sony PlayStation 2. It was designed to determine the participant’s speed by cycling cadence, and faster pedaling was associated with faster movement in the virtual world on screen. The participants were allowed to select videogames to play while cycling and were permitted to switch games during the exercise sessions. There was no nutritional intervention or parental involvement. The participants were weighed in a lab at the end of the sessions.

In the study by Maddison et al [18], participants assigned to the intervention received a Sony PlayStation EyeToy, a dance mat, and a selection of active videogames (eg, Play3, Kinetic, Sport, and Dance Factory), from which they could choose 5 different games. The motion camera placed a picture of the gamer on the screen, which the gamer then interacted with. The participants were asked to achieve the recommended 60 minutes of moderate-to-vigorous physical activity on most days of the week by supplementing periods of inactivity with active videogame play and substituting periods of traditional nonactive videogame play with the active version. There was no nutritional intervention or parental involvement. The participants were weighed at a central location (at baseline, and 12 and 24 weeks) by the researchers.

Oh et al [19] created an intervention using the Super Kids Adventure game that provides upper extremity stretching, lunging, boxing, side-bending, squatting, and arm and jumping exercises. They asked participants to perform activities for 30 minutes 5 times per week at an intensity of about 4-6 metabolic equivalents of task. There was no intervention regarding nutrition. The participants could select various characters to play, and there was real-time visual and auditory feedback given by the game. When the participants performed the correct posture for any of the exercises, the character moved and scored. There was an alarm function that reminded the participants to exercise regularly. They were compensated according to the quantity of training they performed and maintained to progress. There was no parental involvement. The participants were weighed after all the sessions.

Staiano et al [20] created an intervention that consisted of 30-60 minutes of active videogame play every school day. The study included a group that performed the activity in a cooperative way (earn the most points and expend the most calories as a team) and another that performed it in a competitive way (compete against their opponent to earn the most points and expend the most calories). There was no nutritional intervention. During the sessions, the coordinators encouraged completion of each daily exergame routine through periodic verbal reinforcement. There was no parental involvement. Weight was measured at the school-based wellness clinic.

Staiano et al [21] assessed an intervention that consisted of three 1-hour exergaming dancing sessions per week. The participants were allowed to self-select the games, songs, dance mode, intensity level, and dance partner. Gaming coaches provided ongoing motivation, and gifts were provided throughout the 12 weeks to encourage attendance and motivate participants to exercise at a high level. There was no nutritional intervention or parental involvement. The participants were weighed at clinic visits (baseline and end points).

Staiano et al [22] provided the intervention group with a Kinect and Xbox 360 gaming console, a 24-week Xbox Live subscription, 4 exergames (Your Shape: Fitness Evolved 2012, Just Dance 3, Disneyland Adventures, and Kinect Sports Season 2), and a Fitbit Zip to wear. The participants were encouraged to meet a goal of 60 minutes per day of moderate-to-vigorous physical activity for 3 days a week with a family member or friend. The participants could choose between the 4 active videogames provided. They received 3 challenges each week with increasing intensity, difficulty, and duration. They also had telehealth coaching: meeting with a fitness coach (video chat or exergame console) on a weekly basis for the first 6 weeks and biweekly thereafter with individualized feedback, motivation, and encouragement to meet the goal and help to create solutions to barriers for physical activity. There was no nutritional or parental involvement. The participants were weighed at the clinic (at baseline and end point).

In the study by Chen et al [23], the intervention group received a Fitbit Flex and downloaded an app and a link to the iStart Smart for Teens program to their mobile phone. The Fitbit Flex recorded and tracked their physical activity, sedentary activity, and food intake in a diary. The iStart Smart for Teens online educational program had 8 modules (participants were asked to perform 1 module per week) that focused on nutrition and physical activity. The participants also received instructions by mobile phone or computer and supplementary information and tips by app messages. There was no parental involvement. The participants were weighed in the clinic (at baseline, and 3 and 6 months).

Chew et al [24], Cueto et al [25], and Jelalian et al [26] used the Kurbo intervention, which is a mobile app developed to aid adolescents and their families with weight management through dietary self-monitoring (promotion of the gradual reduction of high-calorie foods over time by using the traffic light diet to categorize foods), physical activity behaviors (recommended 60 minutes of moderate-to-vigorous physical activity each day), and weekly individualized coaching sessions (via video, phone, or text) with feedback. Chew et al [24] asked the participants to weigh themselves at least weekly, and they were weighed at clinic visits (at 3 and 6 months). In the other 2 studies, the participants were weighed at baseline and the end point.

Lei et al [27] created an intervention that consisted of a nutritional program based on calorie restriction with meal replacement and individualized low-calorie meal plans. Physical activity was encouraged but with no specific exercise recommendations. The participants had access to their weight loss progress and snapshots of their current health data and optimal measures of BMI. There was parental involvement. The participants were asked to weigh themselves on a daily basis (wireless scale). 

In the study by Mameli et al [28], the intervention group received a wristband that they were required to use for at least 5 days per week (to measure energy expenditure) and an app that allowed real-time recording of food consumption (to measure energy intake) by asking them to enter the raw foods consumed into the app. It had a visual database of foods and 3 portion sizes (small, medium, and large) for each food. The participants received personalized lifestyle programs based on previous week energy intake (for nutritional recommendations) and expenditure (for physical activity recommendations). There was weekly feedback on the adequacy of the diet and physical activity (compliance with the diet, energy gap, sedentary time, physical activity level, and quality of the diet) via text messages. The participants received suggestions on how to reach each of the 5 goals. Positive feedback was included in the text messages every time a participant reached at least one goal, with specification of the reached goals. There was parental involvement. The participants were weighed at clinic visits (at 1, 2, and 3 months).

Pretlow et al [29] and Vidmar et al [32] focused on sequential withdrawal from problem foods, snacking, and excessive food amounts at meals. The participants had 4 face-to-face group meetings, weekly phone meetings, and text messages 5 days per week. The participants also had access to the mentor’s contact details and peer support through app bulletin boards and “weight loss buddies” (chat). There was a monetary incentive proportional to the completion of the requirements of the study. There was no physical activity intervention or parental involvement. The participants were asked to weigh themselves daily (wireless scale), and they were also weighed during face-to-face meetings.

Pretlow et al [30] developed an intervention similar to that in another study [29], in which they added a motor addiction component that consisted of (1) viewing aversive photos/videos or snapping a rubber band against the wrist to quell eating urges, (2) stress reduction, (3) avoiding triggers, (4) relaxation techniques, (5) competing behaviors, (6) distractions, and (7) distress tolerance. Another motor addiction method was the Worry List, a stress reduction feature that prompted participants to journal their current worries and create an action plan for each worry. The participants received daily notifications to login and answer questions about their eating behavior and if they used any sensory and motor addiction treatment methods (if they reported addictive eating behaviors, the app asked why this had happened and what was the participant’s plan to keep this from happening again). Moreover, they received weekly app prompts to update their worry lists and plans.

Stasinaki et al [31] created individual multi-component behavior changing interventions following Swiss guidelines, including handouts on nutritional education and physical activity, and used the support of the PathMate2 app. The participants had daily challenges (eg, number of steps per day). The app included 2 chat channels: virtual coach (daily interaction, encouraging them to achieve challenges to earn virtual rewards) and human coach (maximum 10 minutes during all interventions). In the app dashboard, the participants could also see their progress. There were 4 on-site visits and 2 remote counseling sessions (via telephone). There was no parental involvement. The participants were weighed during clinical visits (at 6 and 12 months).

Cummings et al [33] gave participants a Fitbit device to track daily goals of >60 active minutes or ≥10,000 steps of exercise (participants could choose which one they wanted to achieve). Weekly goals depended upon the level of activity in the prior weeks. The participants received feedback on daily and weekly progress with daily texts about whether they met their goal the previous day (praising them if they met their goal or reminding them to do so if they did not meet their goal) and received weekly texts about whether they met their weekly goal (praising them if they did and providing encouragement if they did not). These texts also informed adolescents of the amount of incentives earned that week and the goal for the upcoming week. There was monetary reinforcement (debit card once per week). There was no nutritional intervention or parental involvement. The participants were weighed during clinic visits (at baseline and end point).

Hinchman et al [34] used a family-oriented approach and incorporated behavior change strategies to address the behaviors, knowledge, attitudes, and self-efficacy of patients and their parents regarding nutrition and physical activity. In the nutritional intervention, they aimed to increase milk consumption until drinking 4 glasses a day, decrease milk fat until drinking fat-free milk, increase fruit and vegetable servings until eating 5 servings a day, and promote eating breakfast every morning. In the physical activity intervention, they aimed to increase the number of days being physically active for 60 minutes until activity for 5 days a week, decrease sedentary behavior to less than 1 hour per day, and increase the number of steps per week on a pedometer until taking 70,000 steps per week. The participants and their parents had weekly 1-hour group appointments (with a health educator/clinician, nursing staff, and a dietitian or chef) that included pedometer games, interactive learning, competitions (for prizes), cooking demonstrations, and group exercises, and involved defining the weekly goals for nutrition and physical activity. The participants and their parents received the Operation Zero manual (with health education, activities, and recipes). The participants were weighed weekly during the appointments.

In short, the interventions focused on only physical activity in 7 studies, only nutrition in 4 studies, and both in 7 studies. Seven studies had parental involvement, and in 3 studies, participants took their own weights.

Regarding extrinsic motivators, the most used was “motivation” (by coaches, messages, and sessions), followed by “feedback” and “rewards” (that included points and money). In the studies that used videogames as the intervention, the most used motivators were “select games,” “rewards,” and “feedback.” In the studies that used an app as the intervention, the most used motivators were “motivation,” “feedback,” and “rewards.”

### Outcomes

The outcomes related to BMI reported for interventions and controls are summarized in Table 2.

**Table 2:** Outcomes related to BMI reported for participants and controls in the selected studies.

Study and country, and outcome		Intervention group			Control group			Intervention group vs control group		
		Outcome data	*P* value	Outcome data		*P* value	Outcome data		*P* value	
**Adamo et al [17], 2010; Canada**										
	BMI (B^a^ vs E^b^)	35.5 (SD 9.3) vs 35.5 (SD 9.7)	>.05	39.3 (SD 8.9) vs 39.4 (SD 8.9)		>.05	—^c^		—	
	pBMI^d^ (B vs E)	97.8 (SD 1.4) vs 97.5 (SD 1.8)	>.05	97.8 (SD 2.7) vs 97.8 (SD 2.3)		>.05	—		—	
**Maddison et al [18], 2011; New Zealand**										
	BMI (Δ|B vs E)	25.6 (SD 4.1) vs 24.8 (SD 3.6)	—	25.8 (SD 4.3) vs 25.8 (SD 4.2)		—	–0.24 (95% CI –0.44 to –0.04)		.02	
	zBMI^e^ (Δ|B vs E)	1.3 (SD 1.1) vs 1.1 (SD 1.1)	—	1.3 (SD 1.1) vs 1.3 (SD 1.0)		—	–0.06 (95% CI –0.12 to –0.03)		.03	
**Oh et al [19], 2022; Korea**										
	BMI (B vs E)	24.99 (SD 1.35) vs 23.93 (SD 1.83)	.02	25.03 (SD 1.92) vs 24.29 (SD 1.83)		.03	—		—	
**Staiano et al [20], 2012; United States**										
	Weight adjusted to growth curve (B vs E)	CO^f^: 93.93 (SD 26.02) vs 84.74 (SD 14.23); CP^g^: 96.22 (SD 17.92) vs 95.17 (SD 20.94)	—	95.48 (SD 22.72) vs 94.23 (SD 20.88)		—	CO (–1.65, SD 4.52) vs C^h^ (0.86, SD 3.01), *P*=.02; CP (0.04, SD 3.46) did not differ from the others		—	
**Staiano et al [21], 2017; United States**										
	ΔzBMI	–0.002 (SE 0.02)	>.05	0.004 (SE 0.02)		>.05	–0.01 (SE 0.03)		>.05	
	ΔpBMI	–0.1 (SE 0.2)	>.05	0.1 (SE 0.2)		>.05	–0.2 (SE 0.3)		>.05	
**Staiano et al [22], 2018; United States**										
	ΔzBMI	–0.06 (SE 0.03)	—	0.03 (SE 0.03)		—	–0.06 (SE 0.03) vs 0.03 (SE 0.03)		.02	
	Δ%BMIp95^i^	–2.2 (SE 1.1)	—	0.9 (SE 1.1)		—	–2.1 (SE 1.1) vs 0.9 (SE 1.1)		.07	
**Chen et al [23], 2017; United States**										
	BMI (B vs E)	27.37 (SD 3.26) vs 26.93 (SD 3.43)	—	28.35 (SD 4.36) vs 29.18 (SD 3.88)		—	z=–4.37		.001	
	zBMI (B vs E)	1.60 (SD 0.50) vs 1.42 (SD 0.38)	—	1.54 (SD 0.42) vs 1.80 (SD 0.50)		—	z=–4.36		.001	
**Chew et al [24], 2021; Singapore**										
	ΔzBMI	0.045 (SD 0.15; 95% CI –0.024 to 0.114)	.19	—		—	—		—	
**Cueto et al [25], 2019; United States**										
	Δ%BMIp95	4 weeks: –5.4 (95% CI –6.2 to –4.5), *P*<.001; 12-16 weeks: –4.8 (95% CI –5.3 to –4.3), *P*<.001; 24 weeks: –6.9 (95% CI –8.3 to –5.6), *P*<.001; Differences between age groups (*P*=.09): 5-11 years: –5.6 (SD 7.9), 12-14 years: –4.7 (SD 5.9), 15-18 years: –5.2 (SD 5.6)	—	—		—	—		—	
**Jelalian et al [26], 2022; United States**										
	ΔBMI	–0.7 (SD 2.19)	—	—		—	—		—	
	Δ%BMIp95	–4.45 (SD 8.5)	—	—		—	—		—	
**Lei et al [27], 2021; China**										
	ΔBMI	–3.13 (MOE^j^: 0.21)	<.001	—		—	—		—	
	ΔzBMI	–0.42 (MOE: 0.03)	>.001	—		—	—		—	
	Δ%BMIp95	–11.51 (MOE: 0.77)	>.001	—		—	—		—	
**Mameli et al [28], 2018; Italy**										
	ΔBMI-SDS^k^	–0.03 (95% CI 0.14 to 0.09)	—	–0.04 (95% CI 0.16 to 0.08)		—	0.01 (95% CI 0.15 to 0.18)		.87	
**Pretlow et al [29], 2015; United States**										
	Δ%overBMI	–0.051	<.01	—		—	—		—	
**Pretlow et al [30], 2020; United States**										
	ΔBMI	–1.6	—	—		—	—		—	
	ΔzBMI	–0.22	<.001	—		—	—		—	
**Stasinaki et al [31], 2021; Switzerland**										
	ΔBMI-SDS	–0.09 (range –0.4 to 0.4)	.33	–0.16 (range –1.9 to 0.3)		>.05	—		—	
**Vidmar et al [32], 2019; United States**										
	zBMI (Δ|B vs E)	–0.09 (95% CI –0.13 to –0.05)	<.001	2.39 (SD 0.34) vs 2.33 (SD 0.38)		.004	–0.02 (95% CI –0.04 to 0.01)		.32	
	%BMIp95 (Δ|B vs E)	127.17 (SD 21.10) vs 118.83 (SD 22.73)	<.001	136.45 (SD 22.76) vs 132.20 (SD 22.49)		.002	–2.04 (95% CI –4.16 to 0.08)		.06	
**Cummings et al [33], 2022; United States**										
	%BMIp95 (B vs E)	110.2 (SD 11.42) vs 110.46 (SD 10.33)	.80	—		—	—		—	
**Hinchman et al [34], 2006; United States**										
	ΔBMI	1.22 (SD 2.8)	<.05	1.60 (SD 2.29)		<.05	No differences between groups at 1 year		—	
	ΔpBMI	0.22 (SD 1.2)	>.05	0.76 (SD 1.86)		<.05	No differences between groups at 1 year		—	

^a^B: baseline.

^b^E: end point.

^c^Not reported or not applicable.

^d^pBMI: BMI percentile.

^e^zBMI: BMI z-score.

^f^CO: cooperative arm.

^g^CP: competitive arm.

^h^C: control.

^i^%BMIp95: percent over the 95th percentile.

^j^MOE: margin of error.

^k^BMI-SDS: BMI standard deviation score.

Of the 18 studies, 9 [19,20,22,23,25,27,29,30,32] reported a statistically significant decrease in BMI and 9 [17,18,21,24,26,28,31,33,34] did not. One of the studies that reported a statistically significant decrease [20] only showed this decrease in 1 of the 2 intervention arms (the cooperative arm).

Among the 9 RCTs, 4 [19,20,22,23] reported a statistically significant decrease in BMI and 5 [17,18,21,28,31] did not. The clinical trial with controls [32] reported a statistically significant decrease in BMI. Among the 3 clinical trials without controls, 2 [29,30] reported a statistically significant decrease in BMI and 1 [33] did not. Among the 4 cohort studies, 1 [25] reported a statistically significant decrease in BMI and 3 [24,26,34] did not. The observational study [27] reported a statistically significant decrease in BMI.

Of the 18 studies, 15 had a follow-up of ≤6 months, and among these, 9 [19,20,22,23,25,27,29,30,32] showed a statistically significant decrease in BMI and 6 [17,18,21,24,28,33] did not. The 3 studies that had a follow-up higher than 6 months [26,31,34] did not report a statistically significant decrease in BMI.

Among the 10 studies that used an app as an intervention, 6 [23,25,27,29,30,32] reported a statistically significant decrease in BMI and 4 [24,26,28,31] did not. Among the 6 studies that used a videogame as an intervention, 3 [19,20,22] reported a statistically significant decrease in BMI and 3 [17,18,21] did not. The other 2 studies [33,34] that used different interventions did not report a statistically significant decrease in BMI.

The only study restricted to the postmenarcheal period did not report a statistically significant decrease in BMI [21].

Regarding the analysis of extrinsic motivators, of the 2 studies that used “avatar” as a motivator, 1 [19] reported a statistically significant decrease in BMI and the other [18] did not. Of the 6 studies that used “challenges” as a motivator, 2 [22,27] reported a statistically significant decrease in BMI and 4 [28,31,33,34] did not. Of the 10 studies that used “feedback” as a motivator, 4 [19,22,25,27] reported a statistically significant decrease in BMI and 6 [17,24,26,28,31,33] did not. Of the 13 studies that used “motivation” as a motivator, 6 [22,23,25,29,30,32] reported a statistically significant decrease in BMI, 6 [21,24,26,28,31,33] did not report a decrease, and 1 [20] reported a statistically significant decrease in the cooperative arm but not in the competitive arm. Of the 5 studies that used “peer-support” as a motivator, 3 [29,30,32] reported a statistically significant decrease in BMI, 1 [34] did not report a decrease, and 1 [20] reported a statistically significant decrease in the cooperative arm (the only arm that had peer-support). The 2 studies that used “reminders” as a motivator [19,30] reported a statistically significant decrease in BMI. Of the 9 studies that used “rewards” as a motivator, 4 [19,29,30,32] reported a statistically significant decrease in BMI, 4 [21,31,33,34] did not report a decrease, and 1 [20] reported a statistically significant decrease in the cooperative arm. Among the studies, 5 used “money incentives” (3 [29,30,32] reported a decrease in BMI and 2 [33,34] did not) and 3 used “points” (2 [19,32] reported a decrease in BMI and 1 [20] reported a statistically significant decrease in the cooperative arm but not in the competitive arm). Of the 4 that used “select games” as a motivator, 1 [22] reported a statistically significant decrease in BMI and 3 [17,18,21] did not.

According to the number of extrinsic motivators applied, 1 study [23] only applied 1 motivator (motivation), and it reported a statistically significant decrease in BMI. Of the 7 studies that applied 2 extrinsic motivators, with 1 being the competitive arm in the study by Staiano et al [20], 2 studies [25,27] reported a statistically significant decrease in BMI and 5 [17,18,20,24,26] did not. Of the 6 studies that applied 3 extrinsic motivators, with 1 being the cooperative arm in the study by Staiano et al [20], 3 studies [20,29,32] reported a statistically significant decrease in BMI and 3 [21,28,34] did not. Of the 5 studies that applied 4 extrinsic motivators, 3 [19,22,30] reported a statistically significant decrease in BMI and 2 [31,33] did not.

### Analysis of Studies That Compared the Impact of the Intervention With Controls

Among the 8 studies that compared the impact of the intervention with controls, 4 [20,22,23,32] reported a statistically significant decrease in BMI (3 RCTs and 1 clinical trial with controls) and 4 [18,21,28,34] did not (3 RCTs and 1 cohort study). 

Among 7 studies [18,20-23,28,32] that had a follow-up of ≤6 months, 4 reported a decrease in BMI and 3 did not, and 1 study that had a follow-up of >6 months [34] did not report a decrease in BMI. 

Three studies [23,28,32] used an app as an intervention (2 reported a decrease in BMI and 1 did not), 4 [18,20-22] used a videogame (2 reported a decrease in BMI and 2 did not), and 1 [34] used Operation Zero (did not report a decrease in BMI). 

One study [18] used “avatar” as a motivator (did not report a decrease in BMI), 3 [22,28,34] used “challenges” (1 reported a decrease in BMI and 2 did not), 2 [22,28] used “feedback” (1 reported a decrease in BMI and 1 did not), 6 [20-23,28,32] used “motivation” (3 reported a decrease in BMI, 2 did not report a decrease, and 1 reported a decrease only in the cooperative arm), 3 [20,32,34] used “peer-support” (2 reported a decrease in BMI and 1 did not), 3 [20,21,32] used “rewards” (1 reported a decrease in BMI, 1 did not report a decrease, and 1 reported a decrease only in the cooperative arm), 2 [21,22] used “select games” (1 reported a decrease in BMI and 1 did not).

One study [23] only applied 1 motivator (reported a decrease in BMI), 2 (with 1 being the competitive arm) [18,20] applied 2 motivators (both reported no decrease in BMI), 5 (with 1 being the cooperative arm) [20,21,28,32,34] applied 3 motivators (2 reported a decrease in BMI and 3 did not), and 1 [22] applied 4 motivators (reported a decrease in BMI).

## Discussion

This review aimed to systematically assess the evidence regarding the use of extrinsic motivators for improving BMI in obese or overweight adolescents.

Half of the studies reported a statistically significant decrease in BMI, and the findings were similar in the subanalysis of studies that compared the impact of the intervention with controls. This indicates how varied the impact on BMI is in this population.

None of the studies that had a follow-up of >6 months reported a statistically significant decrease in BMI. This is in line with the literature [37,38] showing no significant effect of financial incentives on weight loss or maintenance in the long term. One hypothesis for this is that extrinsic motivators can work as a boost for initial change, but the effect decreases over time.

According to the intervention, among the studies that used an app as an intervention, 60% (6/10) reported a statistically significant decrease in BMI. The literature [39,40] has limited and mixed evidence on the impact of mobile app use on motivation and goal-setting behaviors and obesity-related outcomes. Regarding the use of a videogame as an intervention, only half reported a statistically significant decrease in BMI. This is in line with another systematic review [41] that found that using only videogames for weight management does not deliver satisfying results.

Regarding extrinsic motivators, among the studies that used “select games,” only 25% (1/4) reported a statistically significant decrease in BMI. To our knowledge, there are no other studies that analyzed this aspect. One hypothesis for the low impact of the extrinsic motivator “select games” is that in most interventions, the games were similar, and thus, adolescents did not feel that they had much control and that it was really their choice. As for “challenges,” only 33% (2/6) of studies reported a statistically significant decrease in BMI. One hypothesis for this is that most of the “challenges” were imposed and not self-chosen, particularly in studies that used an app as the intervention, which could negatively influence the motivation for completion. 

On the other hand, regarding the use of “rewards” as a motivator, among studies that used “money incentives,” 60% (3/5) reported a statistically significant decrease in BMI. This is in line with the literature [38] showing that financial incentives produce significant weight loss during the intervention, but weight is usually regained following cessation of the incentive [37,38]. Moreover, among studies that used “points,” 75% (3/4) reported a statistically significant decrease in BMI. The mechanism of influence appears to be similar to that for “money incentives” as both work as rewards [42]. Among studies that used “peer-support,” 80% (4/5) reported a statistically significant decrease in BMI. On analyzing the study by Staiano et al [20], we can see that this extrinsic motivator has a major impact on the outcome because only the intervention arm with it (cooperative arm) showed a statistically significant difference compared with the control group. This is in line with a meta-analysis [43] showing that peer-support appears to be associated with decreased weight and BMI levels in individuals with overweight and obesity. One hypothesis is that peers can personalize weight control interventions for individuals with overweight or obesity in a way that medical professionals often fail to achieve. With regard to “reminders,” all studies showed a statistically significant decrease in BMI. One hypothesis is that these “reminders” can help adolescents to not forget about their activities and goals.

The number of extrinsic motivators used was not related to the outcome. This may be due to the different definitions applied to each extrinsic motivator between different studies. For example, “motivation” could involve coaches, messages, sessions, or a mixture of these, and thus, the impact could be different.

Among studies where intrinsic motivation was already present, all showed a statistically significant decrease in BMI. Comparing academic performance [44], students with primarily intrinsic motivators had better academic performance than those with primarily extrinsic motivators. Extrinsic motivation can become an essential strategy for only less interesting or enjoyable tasks [44,45]. Therefore, having intrinsic motivation could be more important than having extrinsic motivation for achieving the goal, and extrinsic motivation can be a boost for more boring tasks.

The trends observed in the subanalysis of studies that evaluated the impact of interventions with controls were overlapping with those observed in the general analysis in most cases.

The studies included had many limitations, and the heterogeneities in study design, sample size, follow-up duration, outcomes reported (BMI, BMI z-score, BMI percentile, percent over the 95th percentile, and BMI SD score), outcome analysis (some reported baseline and end point values and others reported variations in outcomes), and extrinsic motivators used make the comparisons and result analyses difficult. Furthermore, studies did not evaluate extrinsic motivators in isolation and did not report the stage of pubertal development among participants; therefore, each study had numerous confounding factors. Finally, most of the studies had a moderate or high risk of bias.

As a limitation of the review process, we could not obtain 3 studies, although we contacted the authors. Therefore, these studies had to be excluded. We also excluded 1 study owing to language limitations (Korean). We could not perform a subanalysis by age or sex as we did not have access to individual data. We only searched 2 databases, and thus, there may be some publication bias and some relevant grey literature may have been missed. Finally, new studies may have been published after April 2023.

Future studies, including RCTs, should be well designed to minimize the heterogeneity of BMI measures and extrinsic motivator definitions. They should also focus on the impact of specific extrinsic motivators on BMI reduction. Studies having a longer duration are also needed to better understand the long-term impact of extrinsic motivators on weight management success.

In conclusion, the extrinsic motivators “reminders” and “peer-support” appear to have high impacts on BMI reduction. Further studies involving specific extrinsic motivator definitions are needed to closely assess their impacts on BMI reduction, including their long-term impacts. Moreover, future studies should evaluate the impacts of extrinsic motivators on BMI according to other important factors such as the level of sexual development.

## Appendix

Multimedia Appendix 1 Search strategy.

Multimedia Appendix 2 PRISMA (Preferred Reporting Items for Systematic Reviews and Meta-Analyses) checklist.

## Abbreviations

PRISMA: Preferred Reporting Items for Systematic Reviews and Meta-Analyses
RCT: randomized controlled trial
