# Supplementary material for: Use of Extrinsic Motivators to Improve the BMI of Obese or Overweight Adolescents: Systematic Review
Source: J Med Internet Res. 2024 Dec 30;26:e57458. doi: 10.2196/57458 (PMC11730235; doi:10.2196/57458)
Supplement: Multimedia Appendix 1 [file jmir_v26i1e57458_app1.docx]

## **Multimedia Appendix 1.** Search strategy.

### PubMed search strategy:

| **Search number** | | **Query** |
| --- | --- | --- |
| 1 | teen*[Title/Abstract] | |
| 2 | youth*[Title/Abstract] | |
| 3 | adolescen*[Title/Abstract] | |
| 4 | juvenile*[Title/Abstract] | |
| 5 | young adult*[Title/Abstract] | |
| 6 | young person[Title/Abstract] | |
| 7 | young individual*[Title/Abstract] | |
| 8 | young people*[Title/Abstract] | |
| 9 | young population*[Title/Abstract] | |
| 10 | young man[Title/Abstract] | |
| 11 | young men[Title/Abstract] | |
| 12 | young woman[Title/Abstract] | |
| 13 | young women[Title/Abstract] | |
| 14 | youngster*[Title/Abstract] | |
| 15 | first-grader*[Title/Abstract] | |
| 16 | second-grader*[Title/Abstract] | |
| 17 | third-grader*[Title/Abstract] | |
| 18 | fourth-grader*[Title/Abstract] | |
| 19 | fifth-grader*[Title/Abstract] | |
| 20 | sixth-grader*[Title/Abstract] | |
| 21 | seventh-grader*[Title/Abstract] | |
| 22 | highschool*[Title/Abstract] | |
| 23 | college*[Title/Abstract] | |
| 24 | secondary school*[Title/Abstract] | |
| 25 | secondary education*[Title/Abstract] | |
| 26 | high school*[Title/Abstract] | |
| 27 | high education[Title/Abstract] | |
| 28 | adolescent[MeSH Terms] | |
| 29 | young adult[MeSH Terms] | |
| 30 | #1 OR #2 OR #3 OR #4 OR #5 OR #6 OR #7 OR #8 OR #9 OR #10 OR #11 OR #12 OR #13 OR #14 OR #15 OR #16 OR #17 OR #18 OR #19 OR #20 OR #21 OR #22 OR #23 OR #24 OR #25 OR #26 OR #27 OR #28 OR #29 | |
| 31 | obesity[MeSH Terms] | |
| 32 | obesity[Title/Abstract] | |
| 33 | overweight[MeSH Terms] | |
| 34 | overweight[Title/Abstract] | |
| 35 | adiposity[MeSH Terms] | |
| 36 | adiposity[Title/Abstract] | |
| 37 | pediatric obesity[MeSH Terms] | |
| 38 | pediatric obesity[Title/Abstract] | |
| 39 | weight change[Title/Abstract] | |
| 40 | weight loss[Title/Abstract] | |
| 41 | weight loss[MeSH Terms] | |
| 42 | weight maintenance[Title/Abstract] | |
| 43 | ("lose"[Title/Abstract] OR "loss"[Title/Abstract] OR "lost"[Title/Abstract] OR "reduction"[Title/Abstract] OR "reduce*"[Title/Abstract] OR "decrease*"[Title/Abstract]) AND ("weight"[Title/Abstract] OR "BMI"[Title/Abstract] OR "body mass index"[Title/Abstract]) | |
| 44 | (maintain*[Title/Abstract] OR maintenance[Title/Abstract] AND (weight[Title/Abstract] OR BMI[Title/Abstract] OR body mass[Title/Abstract])) | |
| 45 | prevent*[Title/Abstract] AND (weight[Title/Abstract] AND (gain*[Title/Abstract] OR increase*[Title/Abstract])) | |
| 46 | #31 OR #32 OR #33 OR #34 OR #35 OR #36 OR #37 OR #38 OR #39 OR #40 OR #41 OR #42 OR #43 OR #44 OR #45 | |
| 47 | gamif*[Title/Abstract] | |
| 48 | game*[Title/Abstract] | |
| 49 | gamification[MeSH Terms] | |
| 50 | telemedicine[MeSH Terms] | |
| 51 | eHealth[Title/Abstract] | |
| 52 | e-Health[Title/Abstract] | |
| 53 | mHealth[Title/Abstract] | |
| 54 | "mobile health"[Title/Abstract] | |
| 55 | "digital health"[Title/Abstract] | |
| 56 | eCoach[Title/Abstract] | |
| 57 | e-Coach[Title/Abstract] | |
| 58 | "electronic coaching"[Title/Abstract] | |
| 59 | app[Title/Abstract] | |
| 60 | apps[Title/Abstract] | |
| 61 | #47 OR #48 OR #49 OR #50 OR #51 OR #52 OR #53 OR #54 OR #55 OR #56 OR #57 OR #58 OR #59 OR #60 | |
| 62 | #30 AND #46 AND #61 | |

### Web of Science search strategy:

(TS=(teen*) OR TS=(youth*) OR TS=(adolescen*) OR TS=(juvenile*) OR TS=(“young person”) OR TS=(“young individual*”) OR TS=(“young people*”) OR TS=(“young population*”) OR TS=(youngster) OR TS=(first-grader*) OR TS=(second-grader*) OR TS=(third-grader*) OR TS=(fourth-grader*) OR TS=(fifth-grader*) OR TS=(sixth-grader*) OR TS=(seventh-grader*) OR TS=(highschool*) OR TS=(college*) OR TS=(“secondary school*”) OR TS=(“secondary education*”) OR TS=(“high school*”) OR TS=(“high education”)) AND

(TS=(obesity) OR TS=(overweight) OR TS=(adiposity) OR TS=(pediatric obesity) OR TS=(weight change) OR TS=(weight loss) OR TS=(weight maintenance) OR TS=( “weight lose” ) OR  TS=(“ BMI lose”) OR  TS=(“body mass index lose”) OR TS=(“ BMI loss”) OR  TS=(“body mass index loss”) OR TS=( “weight lost” ) OR  TS=(“ BMI lost”) OR  TS=(“body mass index lost”) OR TS=( “weight reduction” ) OR  TS=(“ BMI reduction”) OR  TS=(“body mass index reduction”) OR TS=( “weight reduce” ) OR  TS=(“ BMI reduce”) OR  TS=(“body mass index reduce”) OR TS=( “weight decrease” ) OR  TS=(“ BMI decrease”) OR  TS=(“body max index decrease”) OR ((TS=(maintain*) OR TS=(maintenance)) AND (TS=(weight) OR TS=(BMI) OR TS=(body mass))) OR (TS=(prevent*) AND (TS=(gain*) OR TS=(increase*)))) AND (TS=(gamif*) OR TS=(game*) OR TS=(eCoach) OR TS=(e-Coach) OR TS=("electronic coaching"))

We searched the Web of Science core collection: all editions (Science Citation Index Expanded, Social Sciences Citation Index, Arts & Humanities Citation Index, Conference Proceedings Citation Index – Science, Conference Proceedings Citation Index – Social Science & Humanities, Emerging Sources Citation Index, Current Chemical Reactions, Index Chemistry).
